# Development of Photonic Multi-Sensing Systems Based on Molecular Gates Biorecognition and Plasmonic Sensors: The PHOTONGATE Project

**DOI:** 10.3390/s23208548

**Published:** 2023-10-18

**Authors:** Oscar Nieves, David Ortiz de Zárate, Elena Aznar, Isabel Caballos, Eva Garrido, Ramón Martínez-Máñez, Fabian Dortu, Damien Bernier, Beatriz Mengual-Chuliá, F. Xavier López-Labrador, Jens J. Sloth, Katrin Loeschner, Lene Duedahl-Olesen, Natalia Prado, Martín Hervello, Armando Menéndez, Rainer Gransee, Thomas Klotzbuecher, M. Clara Gonçalves, Fahimeh Zare, Ana Fuentes López, Isabel Fernández Segovia, Jose M. Barat Baviera, Jaime Salcedo, Sara Recuero, Santiago Simón, Ana Fernández Blanco, Sergio Peransi, Maribel Gómez-Gómez, Amadeu Griol

**Affiliations:** 1 Nanophotonics Technology Center, Universitat Politècnica de València, Camí de Vera s/n, 46022 Valencia, Spain; oniepan@ntc.upv.es (O.N.); daorde@ntc.upv.es (D.O.d.Z.); 2 Instituto Interuniversitario de Investigación de Reconocimiento Molecular y Desarrollo Tecnológico, Universitat Politècnica de València, Universitat de València, Camino de Vera s/n, 46022 Valencia, Spain; elazgi@upvnet.upv.es (E.A.); micabgom@doctor.upv.es (I.C.); evgarga5@alumni.upv.es (E.G.); rmaez@qim.upv.es (R.M.-M.); 3 Unidad Mixta de Investigación en Nanomedicina y Sensores, Instituto de Investigación Sanitaria La Fe (IISLAFE) Avenida Fernando Abril Martorell 106, 46026 Valencia, Spain; 4 CIBER de Bioingeniería, Biomateriales y Nanomedicina (CIBER-BBN), Instituto de Salud Carlos III, 46022 Valencia, Spain; 5 Multitel, Parc Initialis 2, Rue Pierre et Marie Curie, 7000 Mons, Belgium; dortu@multitel.be (F.D.); bernier@multitel.be (D.B.); 6 Virology Laboratory, Genomics and Health Area, Fundación para el Fomento de la Investigación Sanitaria y Biomédica de la Comunitat Valenciana, FISABIO-Public Health, Generalitat Valenciana, 46020 Valencia, Spain; mengual_bea@gva.es (B.M.-C.); f.xavier.lopez@uv.es (F.X.L.-L.); 7 CIBER de Epidemiología y Salud Pública (CIBERESP), Instituto de Salud Carlos III, 28029 Madrid, Spain; 8 Departament de Microbiologia i Ecologia, Facultat de Medicina, Universitat de València, 46010 Valencia, Spain; 9 National Food Institute, Technical University of Denmark, Kemitorvet B201, DK-2800 KGS. Lyngby, Denmark; jjsl@food.dtu.dk (J.J.S.); kals@food.dtu.dk (K.L.); lduo@food.dtu.dk (L.D.-O.); 10 Asociación de Investigación de Industrias Cárnicas del Principado de Asturias (ASINCAR), Polígono La Barreda, Calle Solelleros 5, 33180 Noreña, Spain; natalia@asincar.com (N.P.); martinhc@asincar.com (M.H.); armandome@asincar.com (A.M.); 11 Fraunhofer IMM, Carl-Zeiss-Str. 18-20, 55129 Mainz, Germany; rainer.gransee@imm.fraunhofer.de (R.G.); klotzbuecher@imm-mainz.de (T.K.); 12 Instituto Superior Técnico, CQE, Avenida Rovisco País 1, 1049 001 Lisboa, Portugal; clara.goncalver@tecnico.ulisboa.pt (M.C.G.); fahimeh.zare@tecnico.ulisboa.pt (F.Z.); 13 Departamento de Tecnología de Alimentos, Escuela Técnica Superior de Ingeniería Agronómica y del Medio Natural, Universitat Politècnica de València, 46022 Valencia, Spain; anfuelo@upvnet.upv.es (A.F.L.); jmbarat@tal.upv.es (J.M.B.B.); 14 Lumensia Sensors S.L., Camí de Vera s/n, 46020 Valencia, Spain; jsalcedo@lumensia.com (J.S.); srecuero@lumensia.com (S.R.); afernandez@lumensia.com (A.F.B.);

**Keywords:** photonics, molecular gates, localized surface plasmonic resonance (LSPR), porous silica, biosensing, microfluidics, respiratory viruses, chemical contaminants

## Abstract

This paper presents the concept of a novel adaptable sensing solution currently being developed under the EU Commission-founded PHOTONGATE project. This concept will allow for the quantification of multiple analytes of the same or different nature (chemicals, metals, bacteria, etc.) in a single test with levels of sensitivity and selectivity at/or over those offered by current solutions. PHOTONGATE relies on two core technologies: a biochemical technology (molecular gates), which will confer the specificity and, therefore, the capability to be adaptable to the analyte of interest, and which, combined with porous substrates, will increase the sensitivity, and a photonic technology based on localized surface plasmonic resonance (LSPR) structures that serve as transducers for light interaction. Both technologies are in the micron range, facilitating the integration of multiple sensors within a small area (mm^2^). The concept will be developed for its application in health diagnosis and food safety sectors. It is thought of as an easy-to-use modular concept, which will consist of the sensing module, mainly of a microfluidics cartridge that will house the photonic sensor, and a platform for fluidic handling, optical interrogation, and signal processing. The platform will include a new optical concept, which is fully European Union Made, avoiding optical fibers and expensive optical components.

## 1. Introduction

In our daily lives, we are exposed to different health threats, such as pathogens (viruses, bacteria, fungi, etc.) [1,2] and hazardous chemicals (mercury, dioxins, lead, etc.) [3,4]. Since these threats are ubiquitous and can pose risks to public health, taking fast and effective actions to identify and mitigate them is required in order to reduce their potential impact on human health and food safety.

Currently, healthcare systems are facing new challenges in diagnosing diseases with similar clinical symptoms caused by different pathogens, like respiratory viruses such as influenza viruses A or B (IVA/IVB), severe acute respiratory syndrome coronavirus 2 (SARS-CoV-2), or respiratory syncytial virus (RSV), which all can cause acute respiratory infections [5]. Despite the resemblance between these infections, they differ in their management and treatment strategies. Therefore, simultaneous on-site detection of different analytes from a single specimen, known as multiplexed point-of-care testing, is of paramount importance for efficient clinical diagnostics [6].

Nowadays, the polymerase chain reaction (PCR) is the gold standard method for virus detection [7]. However, PCR testing has relevant drawbacks, as it requires a long time between the collection of samples and their processing, needs a clinical laboratory with trained staff, and has a high economic cost per test. Also, the traditional PCR is, at best, semi-quantitative. Although there is a PCR technique named quantitative PCR (qPCR) [8], which also offers multiplexed tests, the quantitative determination by qPCR is indirect, providing a relative quantification of the viral load and is normally limited to 2–3 analytes per tube/assay.

The same needs have emerged in the food sector [9], where the food industry is facing the crucial challenge of ensuring that food is safe for consumers (food safety) while maintaining a production process within environmental constraints. For this purpose, analytical control of the levels of chemicals and microbiological contaminants is mandatory to document compliance with maximum levels in the legislation [10]. The problems leading to food safety issues should ideally be identified early in the value chain (ideally in real-time), from raw materials to the final products, allowing the food industry to minimize their impact through corrective actions.

Currently, depending on the contaminant, different methods are used, including immunological techniques (ELISA), culture-based methodologies, and molecular recognition (PCR) for microbial contaminations [11], and different physio-chemical methods depending on the chemical contaminant, being high-performance liquid chromatography (HPLC) and mass spectrometric (MS) techniques, are the most frequently reported [12]. Those methods are typically time-consuming and labor-intensive, and the results are often not available until after several hours or even days. Moreover, the working principles of the methods for different contaminants are often totally incompatible with each other, increasing the effort needed as several separate analyses are required. Consequently, an easy-to-use solution that allows the rapid detection and quantitative analysis of multiple analytes with high sensitivity in a single test, providing reliable results in a faster, easier, and cheaper way than the current state-of-the-art methods, is being highly demanded by the food industry.

**Figure 1:**
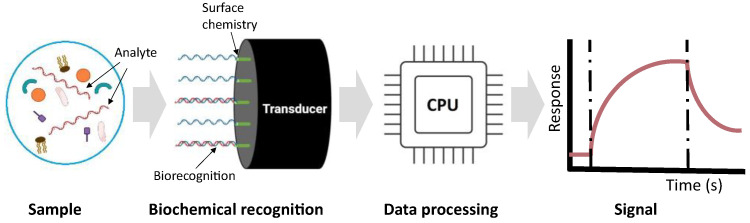
Biosensor mechanism representation.

Biosensor devices have emerged as one of the most relevant diagnostic techniques for different fields [13,14] due to their specificity, ease of mass fabrication, economics, and fast quantitative analysis [15,16]. Figure 1 summarizes the working mechanisms of the biosensors, which include sample collection, biochemical recognition, data processing, and signal obtention [17,18,19].

The current transducers involved in biosensors include different mechanisms, such as electromechanical (potentiometric, amperometric, or calorimetric), piezoelectric, thermal sensors and optical/optoelectronic biosensors (surface plasmon resonance and localized surface plasmon resonance (SPR/LSPR), luminescence, fluorescence, or evanescence waves) [19,20]. Among the abovementioned techniques, SPR/LSPR presents several advantages. First, SPR- and LSPR-based sensors can be fabricated by classical micro/nanofabrication CMOS compatible processes; however, they do not require complex building blocks, such as optical waveguide- or resonator-based biosensors. In addition, SPR/LSPR biosensor-based tests provide a high-sensitivity low limit of detection (LOD), selectivity and cost-effective analyses. Different SPR sensors with a promising sensitivity ranging from 10^−5^ to 10^−8^ RIU have been reported [20]. Even though still limited, there are SPR-based market products (such as a Biacore instrument [21] with a sensitivity between 1 × 10^−6^ to 1 × 10^−7^ RIU). All these SPR developments have a good sensitivity that can satisfy most research requirements; however, there still exist three potential problems that will limit their applications in many fields.

- First, the evanescent field in those basic SPR structures only penetrates the surrounding medium for about 100 nm, and thus, it is very difficult to detect the large target molecules, like cells and bacteria.
- Second, SPR systems, such as the Biacore system, require complex interrogation instruments based on prism coupling, which requires expensive adaptive optics and thermal controls.
- Finally, there are some sensitive SPR biosensors; however, most of them can only detect one analyte. In that sense, the SPR imaging (SPRI) technique is, thus far, the most promising tool for high-throughput multi-analyte detection with a sensitivity of approximately 10^−5^ RIU [18]. The typical SPRI sensor is, however, also based on complex prism coupling instrumentation, in which monochromatic incident light is expanded, passes through a prism, and strikes the interface of the thin film and prism at the coupling angle, exciting a broad area of the sensing surface.

Recent progress in nanostructure fabrication techniques has paved the route toward the development of highly sensitive and label-free optical transducers using the localized surface plasmon resonance (LSPR) of metal nanostructures. The use of such localized surface plasmon resonance (LSPR) structures has been proposed to overcome the described problems of current SPR systems. LSPR structures will increase the detection of smaller targets, as the bulk effect is suppressed, and additionally, will drastically reduce the instrumentation complexity required in SPR systems, avoiding the use of prisms and other complex optics systems [22].

Regarding biochemical recognition, new approaches, such as gated materials, have recently been drawing attention due to their applications in fields such as biomedicine and molecular recognition [14,23]. Gated materials for sensing applications contain nanoporous support loaded with a molecular reporter and are capped with a molecular or supramolecular entity called a molecular gate (also known as a gatekeeper or nanovalve). Gated materials inhibit the delivery of the cargo, yet the presence of certain external stimuli induce a change in the molecular gate, allowing cargo release. Gated materials that are able to respond to chemical, biochemical, and physical stimuli have been reported. Concretely, the use of molecular gates and mesoporous supports has been proven to have meaningful applications in biotechnology and biomedicine, such as drug delivery [24], diagnosis [25,26], or chemical communication [27]. When gated materials are used as probes, the gating mechanism is designed to be controlled by the target species. In this way, once the molecular or supramolecular gates are attached to the outer surface of the porous substrate, upon the external stimulus (target species), the gate is opened, allowing the release of previously entrapped molecules or dyes, which usually act as a reporter.

With the aim of developing an adaptable diagnostics solution based on a very innovative approach, the PHOTONGATE project has emerged [28]. The PHOTONGATE is based on the combination of molecular gates as biorecognition elements [29] and localized surface plasmonic resonance (LSPR) structures as transducers, ref. [30] to detect multiple analytes with high sensitivity. This paper offers an overview of the PHOTONGATE project, focusing on the explanation of the different parts of the sensor and the innovation of the PHOTONGATE approach.

## 2. PHOTONGATE Overall Concept

PHOTONGATE is focused on the detection of diagnostic targets that may cause respiratory infections, such as IVA, IVB, [31,32], SARS-CoV-2 [33], and RSV [34], the detection of two of the main food chemical contaminants in fresh fish (histamine [35] and methylmercury/MeHg [36]), and one of the most virulent foodborne pathogens (*Listeria monocytogenes* [37]).

The PHOTONGATE project will develop a novel biosensor device and a readout platform based on molecular gates and LSPR structures. The PHOTONGATE concept is based on the combination of the following technologies:

1. Molecular gates containing the sensitive probes are able to react to the analytes of interest, viruses, bacteria, and chemical hazards [29,38,39].
2. LSPR structures for sensing—allowing label-free optical detection based on refractive index changes [30,40].
3. Porous nanomaterial [41,42], filled with cargo and closed with molecular gates. Chemical interactions between the targeted analyte and probe will trigger the opening of the gates, allowing the release of the cargo, which is sensed by the LSPR structures, amplifying, in this way, the weak chemical interactions.
4. The polymeric microfluidic system, which allows the flow from a sample to the sensing system [43,44,45].
5. An optical readout platform was produced for this project; this system includes optical emitters and a spectrometer device, which are able to display the sensing signal for up to 12 analytes.

As shown in Table 1, with this approach, PHOTONGATE will provide faster results (around 30 min) with sensitivities comparable to conventional methods for multiple analytes (up to 12 targets). Furthermore, as shown in Table 1, the PHOTONGATE sensing system will provide additional advantages, such as improved cost-effectiveness relations and portability, allowing on-site analysis.

### PHOTONGATE Innovation

PHOTONGATE is innovating on many levels, with the primary developments being the following:

- The PHOTONGATE device will be capable of detecting different chemical and microbial contaminants and viral hazards, being able to work for different fields such as health care and food control. In addition, it requires little training on the part of the personnel since there is a minimum preprocess of the samples, and it will offer an easy reading of the results.
- The LSPR sensors used in the device do not require the use of any fluorescent label (label-free detection).
- The use of the molecular gates mechanism will improve the specificity and selectivity of the biosensors.
- The sensing mechanism, involving the opening of the pores by the probe-receptor interaction, produces a strong change in the refractive index. This mechanism of signal amplification will increase the sensitivity, allowing lower detection limits.
- The analysis will require 30 min or less. Additionally, it evaluates multiple targets with no risk of cross-reactions.
- Fabrication at the wafer scale will ease high-integration and cost-effective devices.
- The portable and easy-to-use readout platform of PHOTONGATE avoids complex components of current SPR commercial systems, enabling them to be used by small clinics, labs, farms, or food producers.

## 3. PHOTONGATE Concept, Overall Design, and Architecture

**Figure 2:**
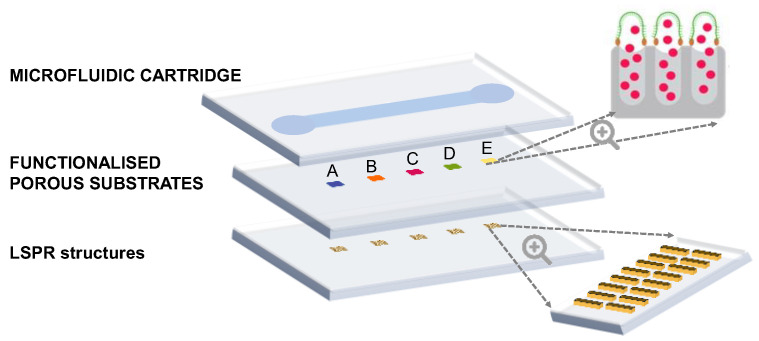
PHOTONGATE sensing module. Scheme of the three technologies integrated into the PHOTONGATE sensing module in the case when five sensors are used Bottom to top: LSPR layer, 5 LSPR sensors, a second layer, which consists of the porous substrate with the pores capped by molecular gates (green) after filling them with the reporter/cargo (red dots), and a third layer which contains the microfluidic channel for exposure to the sensors the sample.

In this section, we present the overall design and specifications of the PHOTONGATE concept. The PHOTONGATE concept consists of two different parts: the sensing module illustrated in Figure 2, and the readout platform described in detail in Section 3.4. The sensing module consists of three parts integrated into a single piece: the LSPR structures, the functionalized porous substrate, and the microfluidic cartridge.

### 3.1. Functionalized Porous Substrate

**Figure 3:**
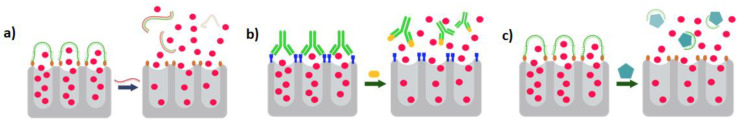
Schematic of a porous substrate filled with dye and closed for three molecular gates containing three different receptors and analyte models to illustrate that adaptability to the analyte of the PHOTONGATE concept. Molecular gate mechanism based on RNA/DNA-DNA complementary sequence (**a**), antigen–antibody (**b**), and contaminant–aptamer (**c**) interactions.

Biosensors obtain their specificity from the biological binding interaction between the analyte and its complementary receptor, which is immobilized onto the transducer surface. In the case of PHOTONGATE, a combination of molecular gates with porous nanomaterials is used for this purpose—being the molecular gates—which confers the specificity to the biosensor. They can be adapted to the desired analyte, working as receptors, and will be used to close the nanostructured porous materials once filled with the selected molecules, as well, as shown in Figure 3. The opening mechanism of the gate is triggered when the attached receptor interacts with the specific analyte; this external stimulus opens the pores, allowing the release of the previously entrapped molecules, giving rise to a strong change in the refractive index inside the porous substrate and producing an amplification of the analyte receptor recognition event, and, consequently, reducing the detection limit. Thus, this technology will offer an enormous potential for developing a multi-analyte system when they are integrated with a photonic technology based on refractive index sensing, such as LSPR structures.

### 3.2. Localized Surface Plasmon Resonance (LSPR) Substrates

**Figure 4:**
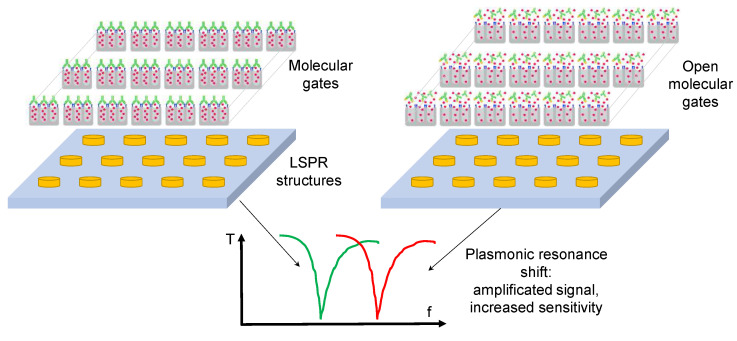
PHOTONGATE transduction based on LSPR structures.

PHOTONGATE will use LSPR substrates as transducers—a periodic array of metallic tailor-made nanostructures fabricated by electron beam lithography (EBL) on a bulk substrate. The light interaction with the LSPR structures gives rise to a plasmonic resonance, which appears as a peak in the optical response signal of the photonic system, as shown in Figure 4. These plasmon peaks are very sensitive to changes in the refractive index of the LSPR environment. When the probes react to the analyte, the molecular gates open, and the entrapped molecules are released; therefore, consequently, the refractive index of the medium surrounding the metallic nanostructures undergoes a change and a shift in the plasmon peak is recorded. The amplified effect obtained by the molecular gates’ mechanism added to the LSPR resonance will allow the detection and quantification of the presence of the target analyte, improving the detection and quantification limits obtained with the current biosensors. Figure 4 shows the proposed PHOTONGATE biosensor mechanisms.

Moreover, the reduced size of the sensors will allow the integration of multiple sensors (each one with the aim of detecting a different target) in a single sensing chip.

### 3.3. Polymeric Microfluidic System

The microfluidic system will be designed to flow the sample that contains the analyte over the biosensing surface (the porous substrates filled with molecules and closed with the molecular gates). It will be properly bonded to the LSPR substrate combined with the biosensing surface, avoiding any possible cross-contamination. The fluidic system will essentially consist of a microfluidic channel having an inlet and an outlet at both ends of the channel, which connects to the sample reservoir and the waste, respectively. The sensor substrate is glued into a cartridge notch, allowing accessibility for the optical interrogation of the sensor spots from the visible up to the near-infrared spectrum.

**Figure 5:**
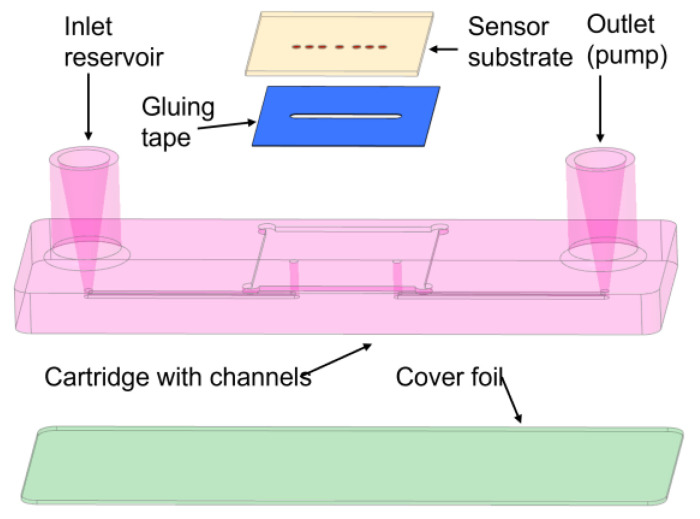
Schematics of the proposed PHOTONGATE cartridge.

With this system, performing a test will only require the insertion of some droplets of the liquid sample into the corresponding inlet reservoir, the insertion of the cartridge into the PHOTONGATE platform, and the start of the assay from the photonic readout platform. If the sample preparation chemicals allow, the cartridge will preferably be made from inexpensive thermoplastics like polypropylene (PP), polystyrene (PS), polymethacrylate (PMMA), or, alternatively, from more chemically resistant plastics like, for example, polyether ether ketone (PEEK). For first-evaluation tests, cartridges can also be made by using rapid-prototyping 3D-printing materials. The inlet reservoir will have typical dimensions below 1 mm, which are compatible with the expected flow rates of several microliters per minute, generated by the connected vacuum pump. The channels will be closed to the outside world using a cover foil and a suitable bonding process to attach it to the cartridge surface surrounding the channels (see Figure 5).

### 3.4. Optical Readout Platform and Sensing Data Analysis/Algorithms

**Figure 6:**
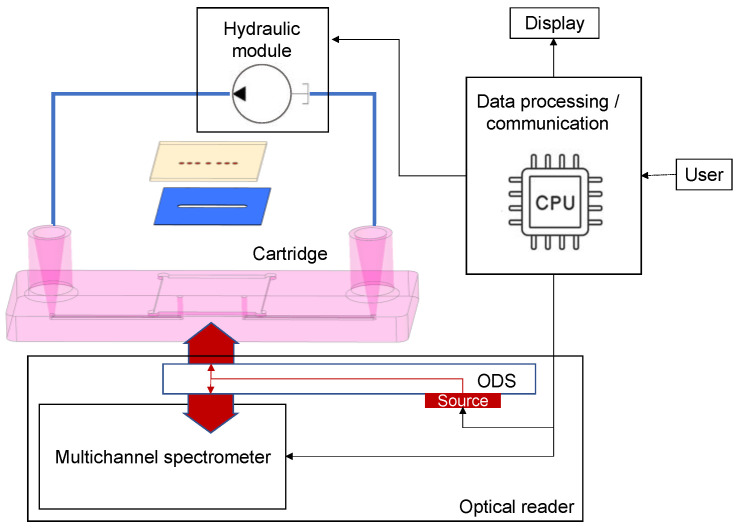
Reader instrument scheme, showing optical readout subsystem, hydraulic module, and embedded processing and connectivity.

The PHOTONGATE readout platform (Figure 6) will automatically carry out the assay once the sensing module is inserted. It is planned to be composed of three subsystems: the optical reader, the hydraulic module, and the embedded data processing and communication module.

**Figure 7:**
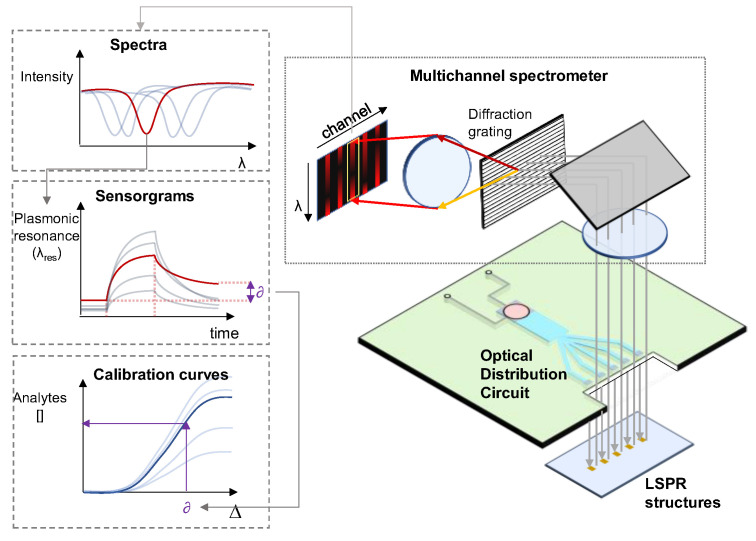
The optical reader concept, based on an optical distribution circuit and a multi-channel spectrometer. Light from each sensor (grey lines) (here only the LSPR layer has been included for the sake of clarity) will be acquired by the multichannel spectrometer.

The hydraulic module will be responsible for flowing the sample over the photonic sensors with fine control of the flow rate. The optical reader will be based on a multi-channel integral field near-infrared (NIR) broadband spectrometer (610 nm–950 nm) and an optical distribution circuit (ODS). The LSPR sensors will be illuminated by a broadband halogen lamp (360 nm–2600 nm) coupled to the ODS situated in close proximity to the LSPR sensor glass substrate (opposite to the microfluidics flow cell). The spectrometer is based on a 600 lines/mm transmission diffraction grating, a 50 mm focal length doublet for light collection, and a 50 mm focal length camera objective imaging the LSPR sensors on an 11.34 m × 7.13 mm silicon focal plane array. It will be able to measure up to 24 optical spectra at a time (considering an LSPR sensor pitch of 250 µm) with a sub-10-picometer resolution for the resonant feature extractions. Raw spectra will be processed using specifically developed algorithms directly executed on the embedded data processing module (ARMv8). Spectral features, as a function of time (sensorgrams), will be shown in parallel on the platform’s touch screen. The automated and embedded analysis of the sensorgrams will result in the quantitative concentrations of the target analytes in less than 30 min after the insertion of the cartridge on the platform. A schematic of the optical reader is shown in Figure 7.

### 3.5. Sensing Data Analysis/Algorithms

The raw data obtained with the readout module will be processed by the mass data processing and communication module. The concentration of each target will be determined through the acquisition of a pair of measurements: (i) its own mathematical model, where the signals acquired by the readout platform will be the input, and the prediction of the parameter will be the output; and (ii) the reference analytical method.

For the construction of the models, two types of models will be used: partial least squares methods (PLS) and neural networks (NN). These two methods are the most used algorithms for the adjustment of linear (PLS) and nonlinear (NN) functions. To improve the performance of the mathematical model, pretreatments of raw data will be applied (smoothing, normalization, Savitzky–Golay, MSC, SNV, detrend, etc.), as well as variable selection methodologies (genetic algorithms). Combinations of previous methods with the two considered regression methods will be analyzed to achieve the final mathematical model.

As a result, using specifically developed algorithms and software, the spectral features will be converted into a quantitative response of the target concentrations that will be plotted as an individual graphical representation over time (sensorgrams). Through this system, quantitative results will be provided 30 min after introducing the sample to the platform.

## 4. Validation

Since this technology will be cost-effective and portable, it can be implemented in different scenarios, such as small farms, food manufacturing industries, clinics, or primary health care centers. A preliminary validation of the PHOTONGATE concept will be performed in food (fish control) and health safety (respiratory infection) applications in a laboratory with pattern samples for proper calibration and adjustments. Finally, the platform will be placed in the research facilities of two different EU countries (Spain and Denmark) for their respective validation against the golden standard techniques used in their respective areas. Additionally, the PHOTONGATE system will be validated in real processing food scenarios and can be emulated for a full food analysis.

Before the final validation, an initial test and troubleshooting phase will be carried out. In the case of target pathogen (*L. monocytogenes*) and chemical contaminants (MeHg and histamine), the complete PHOTONGATE system will be tested with real samples (including incurred material as well as spiked samples). Therefore, in the cases of *L. monocytogenes*, the selected food samples will be inoculated with known counts of the pathogen and *L. innocua* strain. In the case of MeHg and histamine, different amounts will be added to the samples in low (close to limit of quantification), medium (close to legal limits), and high (above legal limits) concentration ranges and the recovery will be used to evaluate the method’s performance.

A preliminary evaluation of the analytical measurement range and other critical performance characteristics will be performed in relation to regulatory guidelines and international guidelines. The obtained results will be compared to the results obtained with the reference analytical methods for each target contaminant: (i) *L. monocytogenes*: the count will be carried out through the validation protocol AFNOR, i.e., in a chromogenic agar culture medium (ALOA), detecting all bacteria related to the genus Listeria through the ß-glucosidase activity determination (*L. monocytogenes* is differentiated as a result of the formation of a phospholipid precipitation halo derived from phospholipase activity); (ii) MeHg: selective extraction (principles in EN17266 [46] and Cressy et al., 2020 [47]) followed by ICPMS detection and the HPLC-ICPMS method; and (iii) histamine: the fluorometric method with HPLC/HPLC-UV (EU regulation Reg. (EC) 2073/2005).

The final validation of the readout platform in the relevant environments will be carried out by using the system for the analysis of real samples. In the case of the detection of respiratory viruses, virus stocks produced in cell culture will be used. This represents a higher titer of the pathogens in a relatively simple matrix (tissue culture media) for the initial optimization/testing of the molecular gates. Later on, the PHOTONGATE system will be tested by using real human nasopharyngeal/nasal and oropharyngeal samples collected in the virus transport medium (VTM), which will be tested in parallel to a gold-standard reference test (the multiplex-RT-PCR virus screening platform). A panel of 50 positives and 50 negatives for each of the four viruses (IVA, IVB, RSV, and SARS-CoV-2) to assess the sensitivity, specificity, and cross-reactivity of each viral analyte will be used. In addition, an additional panel of 50 samples with mixed viral infections to assess the sensitivity for multiplex detection will be analyzed. Finally, 100 samples, collected prospectively using the real-time RT-PCR gold standard and the PHOTONGATE system in parallel, will be assessed.

In parallel, the PHOTONGATE technology will be validated for food samples (different fish samples). For this purpose, the quantitative microbiologic methods’ (*L. monocytogenes*) accuracy (or recovery) and precision under reproducibility conditions will be determined, allowing us to estimate the method’s uncertainty. In the case of the histamine and MeHg contaminants, the parameters to be evaluated will be accuracy and precision under reproducibility conditions, linear range, limit of detection and quantification, and measurement uncertainty. Concerning the quantitative methods, the validation will be carried out along the whole defined range, i.e., high, medium, and low concentrations. Furthermore, whenever possible, reference materials with certified target values for the contaminants will be used during the validation process.

## 5. Conclusions

In this paper, we describe the preliminary architecture and design of the PHOTONGATE biosensor device. It is a modular concept based essentially on two validated technologies: molecular gates technology (the biological receptor) and LSPR structures (the transducer element based on refractive index sensing), which are both integrated into a microfluidic module or cartridge. Together with the readout module or platform, this concept will allow the label-free optical detection of multiple analytes with high sensitivity, also improving the time analysis and cost-effectiveness of the current sensing methods, as well as offering an easy readout of the results. Moreover, it will be an easy-to-use technology, which will require little training on the part of the personnel since there is no need to preprocess the samples. Based on this innovative approach, PHOTONGATE will develop an adaptable biosensor device for the detection of a variety of analytes with applications in the food and healthcare sectors.

Finally, the PHOTONGATE system will be validated using internationally recognized methods to assess the relevant method’s performance characteristics. Trueness, repeatability, reproducibility, detection limits, linearity, analytical measurement range, as well as reporting range will be assessed using realistic sample material. Specificity will be assessed by testing a range of theoretically relevant chemical moieties, individually and in combination. Robustness will be assessed both in relation to the experimental conditions and matrix variability (species type, sample conditions, etc.).

## Figures and Tables

**Table 1:** PHOTONGATE system advancements regarding traditional techniques.

PHOTONGATE System Advancements			
Feature		Current Systems	PHOTONGATE System
Meas. range	Histamine	ppm range	ppm range for liquid and solid samples
	MeHg	ppb range	ppb range
	Microbial analysis	20–1000 CFU/g	1 CFU/25 g
	Viral analysis	1–250 viral copies/μL (RT-PCR)	1–5 viral copies/μL
Selectivity (Specificity)		Chemical contaminants: Typical analyte interference caused by the food matrix. Microbial and viral hazards: Specificity, based on specific media in culture-based methods and primer design in PCR.	Increased selectiveness among contaminants, e.g., validated discrimination between close species (inorganic mercury and MeHg) in model system. Specificity based on probe/antibody, ARN/ADN designs.
Analysis time		Several hours/days	30 min for up to 12 targets
Cost-effectiveness		MeHg/histamine: EUR 200–300 per sample Microbial and viral: One analysis from EUR 20 to 100 dep. on target.	EUR 20 per chip for 4 analytes, Platform manufacturing: EUR 7 K (to be used for 10,000 analyses)

## Data Availability

Not applicable.
